# Genomic characterization and fermentation study of the endophyte *Stemphylium* sp. (Aa22), a producer of bioactive alkyl-resorcinols

**DOI:** 10.1371/journal.pone.0323031

**Published:** 2025-09-17

**Authors:** Jorge Rojas López-Menchero, Juan Imperial, Maria Fe Andrés, Carmen Elisa Díaz, Azucena González-Coloma

**Affiliations:** 1 Instituto de Ciencias Agrarias, CSIC, Madrid, Spain; 2 Escuela Técnica Superior de Ingeniería Agronómica, Alimentaria y de Biosistemas (ETSIAAB), Universidad Politécnica de Madrid (UPM), Madrid, Spain; 3 Centro de Biotecnología y Genómica de Plantas UPM-INIA/CSIC, Parque Científico y Tecnológico, UPM Campus de Montegancedo, Madrid, Spain; 4 Instituto de Productos Naturales y Agrobiología, CSIC, La Laguna, Spain; Qassim University, SAUDI ARABIA

## Abstract

The genome of the previously described endophytic fungus *Stemphylium* sp. (strain Aa22) has been sequenced to near completion. Phylogenomic analysis placed strain Aa22 in close proximity to *Stemphylium lycopersici*. Strain Aa22 had been previously reported as the producer of the bioactive alkyl-resorcinol stemphol and derivative stempholones A and B in solid culture on rice. Genome mining for biosynthetic gene clusters (BGCs) identified 38 genomic regions predicted to encode secondary metabolites production. Among them, a single type III polyketide synthase (T3PKS) that shared similarity with other fungal T3PKSs was identified. T3PKSs are responsible for the biosynthesis of alkyl-resorcinols from fatty acyl-CoA substrates. This makes the identified T3PKS gene a likely candidate for stempholone biosynthesis and a target for future manipulation to enhance production of bioactive alkyl-resorcinols. We also studied the production of these compounds in solid rice media and in liquid PDB medium with or without the addition of talcum powder. The highest extract yield was obtained with PDB cultures, and GC-MS analysis revealed the presence of high levels of the bioactive compound stempholone A, along with two unidentified compounds. Addition of talcum powder suppressed stempholone A production and reduced chemical diversity, with accumulation of oleamide. In contrast, the rice solid media fermentation resulted in methylated fatty acids and oleamide, with no detectable stempholone.

## Introduction

Currently, agriculture is facing the global challenge of being productive, efficient, sustainable, and environmentally friendly [1]. Food production is affected by plant diseases and insect pests [2]. To date, plant protection has essentially depended on synthetic pesticides, resulting in negative environmental and health impact. In this context, there has been an increase in the research on new safer bio-pesticides [3,4]. Additionally, stricter pesticide safety regulations make the search for new bio-pesticides a priority [3].

Endophytic fungi are present in tissues or organs of plants without causing apparent damage [5] and contribute to the adaptation of the plant to abiotic and biotic stresses [6,7]. Endophytic fungi are capable of producing a diverse range of bioactive metabolites, including those with insecticidal, antioxidant, antifungal, antiviral, antibacterial, or cytotoxic activities that have potential as biopesticides [8–10]. Thus, fungi represent a promising novel source for the biotechnological production of valuable active compounds [11–13].

Most fungal endophytes are traditionally identified morphologically and genetically through analysis of the internal transcribed spacer regions of nuclear ribosomal DNA (ITS1-5.8S-ITS2 rDNA). However, with the advent of powerful, affordable next-generation sequencing (NGS) methods, these are being increasingly used to study endophytes for taxonomic identification along with approaches such as metabolomics, metagenomics, proteomics and bioinformatics [14]. Functional genomics sequencing of fungal endophytes involves a comprehensive analysis of their genetic material to understand the roles of specific genes and metabolic pathways in plant-microbe interactions. Therefore, functional genomics can reveal the potential of microorganisms in biotechnological applications [15–17]

The study of the endophytic biodiversity from medicinal plants has resulted in the identification of bioactive compounds. One example is wormwood (*Artemisia absinthium*), where endophytic bacteria are a remarkable source of antimicrobial and anticancer compounds such as 2-aminoacetophenone, 1,2-apyrazine-1,4-dione, phenazine and 2-phenyl-4-cyanopyridine [18]. Furthermore, in a recent study, our group showed that the fungal endophytic strain Aa22, isolated from *A. absinthium* and previously identified as *Stemphylium solani,* was able to yield an ethyl acetate extract from the solid rice fermentation that had antifeedant properties against *Myzus persicae* aphids [19]. The bioguided fractionation of this extract led to the isolation of the alkyl-resorcinols stempholone A, stempholone B and stemphol. All these compounds were aphid antifeedants, and stempholone A was also moderately nematicidal. These results highlight the potential of the endophytic strain Aa22 as a biotechnological source of natural product-based biopesticides.

Fungal fermentation conditions significantly influence the production of bioactive compounds of interest. Submerged fermentation (SmF) and solid-state fermentation (SSF) are the most widely used methods, both with advantages and disadvantages [20–22]. SSF has the potential to reduce costs and energy consumption [20]. However, achieving stable fermentation conditions is more challenging due to limited control over parameters such as pH, oxygenation, and moisture [23], which complicates the scalability of the process. On the other hand, SmF offers advantages such as higher productivity and yields [24,25], and allows for easier and more reproducible control and measurement of fermentation parameters [26]. However, in SSF, the development of mycelial clumps or pellets reduces the effective transfer of oxygen and nutrient resources in the liquid phase [27]. In this context, semi-solid-state fermentation (Semi-SSF) is a hybrid method that utilizes both an inert solid support in a determined volume of culture medium [28]. Semi-SSF allows the production of secondary metabolites in high quantities under controlled conditions [29,30] by reducing the formation of fungal agglomerates [31], thus leading to more efficient substrate consumption, greater oxygen transfer, and increased productivity of the fungus. Among Semi-SSF techniques, microparticle-enhanced cultivation (MPEC) with talcum powder or aluminum oxide [32] is widely used. When microparticles are added to culture media, some fungi tend to attach to them, inducing changes in the metabolism and as a result, the pattern of metabolites production can be modified [29,30,33,34].

The objectives of this work include: (a) The genomic characterization of strain Aa22 in order to obtain a clear-cut taxonomic placement for the strain along with insights into the genetic determinants of its ability to produce bioactive alkyl-resorcinols. (b) The study of the production conditions of the bioactive compounds by means of several fermentation conditions, including solid (rice) and PDB liquid culture media with or without the addition of the microparticle-enhanced cultivation (MPEC) talcum as a potential inductor of metabolic changes. The ethyl acetate extracts obtained were analyzed for their chemical composition searching for targeted bioactive alkyl-resorcinols previously described in this strain.

## Materials and methods

### Biological material

The endophytic fungus Aa22 was isolated from *Artemisia absinthium* leaves. This strain was previously identified as *Stemphylium solani* and deposited in the Spanish Type Culture Collection (CECT 20941) [19].

### DNA extraction and size selection

High molecular weight fungal DNA was isolated using a modified cetyltrimethylammonium bromide (CTAB) method [35]. Briefly, portions of approximately 150 mg of fungal mycelium grown in PDB medium for 3 days were washed with distilled water, centrifuged at 16,000 x g 1 min, and the pellet was ground to a fine powder in liquid nitrogen. The powder was mixed with 500 μL of CTAB buffer and 1.2 μL of 2-mercaptoethanol, followed by the addition of 2 μL of RNase A (10 mg/mL). After incubation at 65°C for 30 min with periodic mixing, proteins and lipids were removed by extraction using a chloroform-isoamyl alcohol mixture (24:1), with centrifugation at 17,300 x g for 10 min. The aqueous phase was transferred, and DNA was precipitated with 550 μL of cold isopropanol and 1/10 volume of 3M sodium acetate, followed by incubation at −20°C for 15 min. The DNA was pelleted by centrifugation at 9,500 x g for 10 min at 4°C, washed sequentially with 100% and 70% ethanol, air-dried and resuspended in EB buffer. DNA was purified using a size selection buffer to remove fragments under 10 kb [36], as follows. A 60 μL portion of DNA suspension was mixed with an equal volume of the size selection buffer. The mixture was centrifuged at 10,000 x g for 30 min at room temperature to pellet high-molecular-weight DNA, and the supernatant was carefully removed. The pellet was washed twice with 200 μL of 70% ethanol, centrifuging for 2 min at 10,000 g after each wash. The air-dried pellet was resuspended in EB buffer. DNA concentration and integrity were assessed using UV absorbance (Nanodrop) and fluorometry (Qubit). DNA purity was assessed based on 260/280 and 260/230 ratios, ensuring suitability for downstream applications.

### Genome sequencing, annotation and analysis

PacBio DNA HiFi libraries were prepared following the standard protocol and sequenced by Novogene (Cambridge, UK) on the Revio platform (PacBio, Menlo Park, CA). The genome was assembled and polished using Flye (v. 2.9); [37]. Genome sequences were annotated with Funannotate (v. 1.8.7) [38]. Biosynthetic gene clusters (BGCs) were identified using antiSMASH fungal version (v.8.0.1; https://fungismash.secondarymetabolites.org/) [39] with detection strictness set to “relaxed” and all extra features enabled. Taxonomic profiling was performed with the UFCG pipeline (v. 1.0.6) [40], which extracts a predefined set of 61 conserved fungal marker genes from the genome sequences. These markers were aligned and subsequently used to infer phylogenetic relationships. The phylogenetic tree was constructed with IQ-TREE (v2.3.1) [41] using the JTT (Jones–Taylor–Thornton) substitution model, incorporating empirical amino acid frequencies (F), a proportion of invariable sites (I), and a gamma distribution (G) to account for rate variation across sites. Branch support was evaluated through 1,000 bootstrap replicates. The resulting tree was visualized using FigTree (v1.4.4) [42]

Genome assembly quality was assessed with QUAST (v.5.2.0) [43], and completeness was evaluated with BUSCO (v.5.3.2) for *Ascomycota* and *Dothideomycetes* [44]. Reference genomes and sequences used for identification were retrieved from publicly available data at NCBI (https://blast.ncbi.nlm.nih.gov/datasets/genome). Geneious (v. 9.1.8; [45] was used to build an Aa22 genomic database and to perform BLAST searches against our genome data. Protein sequences were aligned in NCBI (https://blast.ncbi.nlm.nih.gov/Blast.cgi). Functional domain annotation of protein sequences was conducted using InterProScan (https://www.ebi.ac.uk/interpro/search/sequence/).

*Stemphylium* sp. Aa22 whole genome shotgun project has been deposited at DDBJ/ENA/GenBank under the accession JBQVVG000000000. The version described in this paper is version JBQVVG010000000.

### Fermentations

For solid-state fermentation (SSF) cultures were grown on rice grains. Firstly, inocula were grown on small Petri dishes (4 cm) with PDA medium (Difco, Maryland, USA). From these plates, sterile, 250-ml Erlenmeyer flasks containing 100 g of rice (commercial brand SOS Redondo, San Juan de Aznalfarache, Seville, Spain) and 50 ml of distilled water were inoculated with 6 mycelium fragments (1 cm^2^ each). Flasks were incubated at 25 °C in darkness for 7, 14, or 20 days (four replicates per time point).

For liquid-state fermentation PDB (BD-Difco, Sparks, MD) medium, with or without a MPEC physical support (talcum powder) was used. Cultures started from PDA plates. Sterile distilled water (10 mL) was added to each fully-grown PDA plate, its surface scraped with a spatula to obtain a mycelium suspension, and 2.5 mL of the suspension transferred to 4 500-mL Erlenmeyer flasks containing 100 mL PDB medium. Flasks were incubated in a rotary incubator (120 rpm; 25ºC) in darkness, for 3 days. 20-mL portions of this preinoculum were used to inoculate 4 x 500-mL Erlenmeyer flasks containing 300 mL of PDB with or without talcum powder, and flasks were incubated under the same conditions for 7, 14, or 20 days (two replicates per time point). For MPEC cultures, talcum powder (Fisher Chemical, particle size 45 μm) was added at 6.6 g-L^-1^.

### Extract preparation

Mycelia were separated from culture media by paper filtration using a Büchner funnel. For SSF, filtered media were Soxhlet extracted (hexane followed by ethyl acetate). For liquid fermentations, sequential liquid-liquid extractions with hexane (to remove lipids) and ethyl acetate were carried out. After extraction, anhydrous Na_2_SO_4_ was added to the organic phase to remove any water traces, then filtered and rotary-evaporated at a reduced pressure.

Yields for liquid-liquid extractions were calculated as follows:


$$Y\;(\%)=\;\left(\frac{Final\;extract\;mass\;(mg)}{Initial\;aqueous\;volume\;(mL)}\;x\text{ 100}\right)$$

Where the final extract mass is the weight of the extract after solvent evaporation, and the initial aqueous volume is the amount of culture medium used for extraction.

### Gas Chromatography-Mass Spectrometry (GC-MS)

Analysis were conducted using a Shimadzu GC-2010 gas chromatograph equipped with a 30 m × 0.25 mm i.d. capillary column (0.25 μm film thickness, 95% Dimehyl- 5% diphenylpolisiloxane, Teknokroma TRB-5) coupled to a Shimadzu GCMS-QP2010 Ultra mass detector (electron ionization, 70 eV). Samples (1 μl) were injected with a Shimadzu AOC-20i autosampler. Working conditions were as follows: split ratio (20:1), injector temperature 300ºC, temperature of the transfer line connected to the mass spectrometer 250 °C, initial column temperature 110 °C, then heated to 290 °C at 7 °C/min, and a full scan was used (m/z 35–450). Electron ionization mass spectra and retention data were used to assess the identity of compounds by comparing them with those found in the Wiley 229 and NIST 17 Mass Spectral Database and injected pure compounds (stemphol and stempholones A and B) isolated from Aa22 [19]. All extracts (4 μg/μl) were dissolved in 100% dichloromethane for injection. Blank ethyl acetate extracts of all media were analyzed as controls.

## Results

### Genome sequence and phylogenomics of Aa22

Long genome sequence reads were obtained from high quality Aa22 DNA. A total of 5,160 Mb of HiFi sequence data was generated and assembled into 24 contigs, with a total genome size of 34.1 Mb and a mean coverage of 145x (Table S1 in S1 File). The average GC content of 50.8%, and N50 and N90 values for this assembly were 1.79 Mb and 1.13 Mb respectively, indicating a high-quality assembly. The near-completeness of this genome sequence was confirmed by BUSCO analysis. For phylum Ascomycota, 1,647 complete BUSCOs were found out of 1,706 (96.5%), of which 1,644 were single-copy and 3 were duplicated. Twelve more were fragmented, and 47 (2.8%) were missing. For class Dothideomycetes, 3,625 BUSCOs were detected out of 3,786 (95.8%), with 3,621 as single-copy, 4 duplicated, 30 fragmented, and 131 (3.5%) missing..

**Fig 1:**
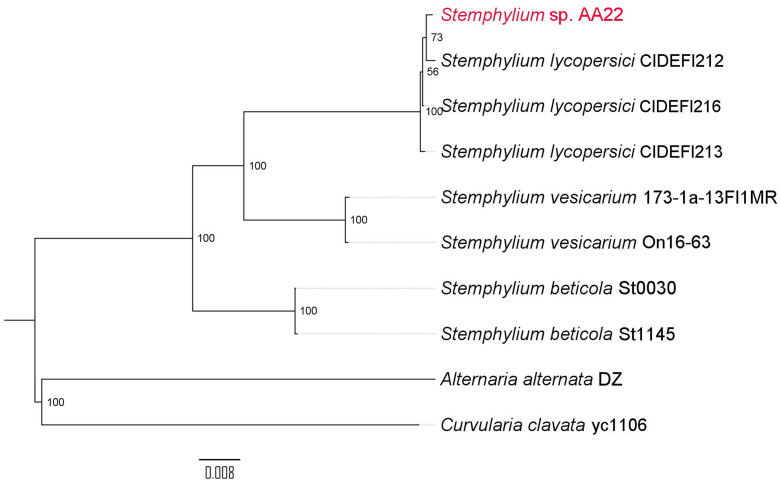
Phylogenomic tree of *Stemphylium* species. UFCG conserved fungal markers were used for tree inference. *Alternaria alternata* DZ and *Curvularia clavata* yc1106 were used as outgroups. Genomes were retrieved from the NCBI database (S2 Table in S1 File). Figures indicate the % bootstrap support (1,000 replicates) for each node in the tree.

Availability of the near-complete Aa22 genome sequence allowed us to reappraise its taxonomic placement. Strain Aa22 had been classified as *Stemphylium solani* on the basis of the identity of a 580 bp sequence containing an incomplete ITS1-5.8S rRNA-ITS2 region with that of a *S. solani* strain [19]. A BLASTN search of this sequence against the core-nucleotide BLAST database revealed 46 sequences with 100% identity. Only two of them were *S. solani* (out of 126 *S. solani* sequences in the database), 41 were *S. lycopersici* (out of 201 *S. lycopersici* sequences in the database), and 3 were unclassified. This raised the possibility that the two *S. solani* strains whose sequences were 100% identical to that of Aa22 may have been misclassified and that Aa22 was indeed a *S. lycopersici* strain. The Aa22 genome data, together with those of all available *Stemphylium* genomes at NCBI, were used for phylogenomic reconstruction (Fig 1). Strain Aa22 clustered with three *S. lycopersici* genomes (strains CIDEFI212, CIDEFI213, and CIDEFI216) in a strongly supported clade (bootstrap value = 100), indicating close evolutionary relatedness to this species. Within this clade, Aa22 was more closely related *to S. lycopersici* CIDEFI212, with relatively high consistency (bootstrap value = 73). This clade was clearly distinct from other *Stemphylium* species for which genome sequences are available, such as *S*. *vesicarium* and *S. beticola*, which formed separate groups within the phylogeny (Fig 1). However, no *S. solani* genomes have been completely sequenced to date. Taken together, the available evidence did not allow assigning strain Aa22 to either *S. solani* or *S. lycopersici*, two phytopathogenic species that appear to be closely related. Thus, the strain was accordingly designated as *Stemphylium* sp. Aa22 until more genomes become available.

### Biosynthetic potential in Aa22 genome

**Fig 2:**
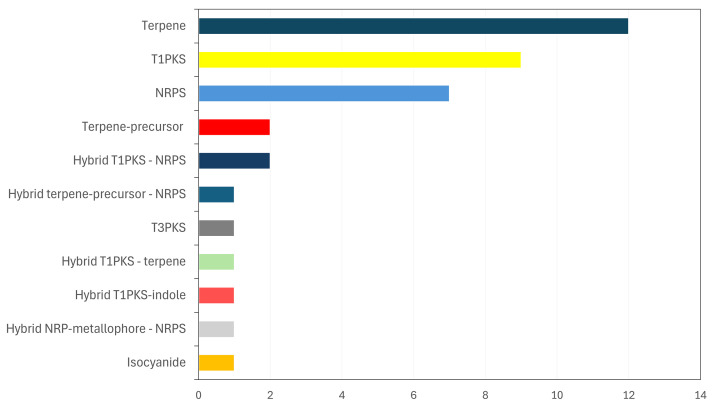
BGCs associated with *Stemphylium* sp. isolate Aa22. Their location and specific characteristics are shown in Table S3 in S1 File.

Analysis of the Aa22 genomic sequence with fungal antiSMASH identified 38 gene coding regions associated with the production of secondary metabolites (Fig 2, Table S3 in S1 File). Among these, 14 regions displayed a varying degree of similarity to BGCs identified in other fungi, including 7 with low similarity, 3 with medium similarity and 4 with high similarity. In contrast, the remaining 24 regions showed no clear match to known biosynthetic pathways. A total of 12 clusters were predicted to encode terpene biosynthesis, two were identified as terpene-precursor related, and one region combined terpene-precursor and NRPS characteristic. Nine regions were linked to polyketide synthesis, specifically type I PKSs (T1PKS), and one to type III PKS (T3PKS). Seven clusters were classified as non-ribosomal peptide synthetases (NRPS). Additional clusters included one isocyanide BGC and one hybrid NRP-metallophore/NRPS. Hybrid biosynthetic gene clusters were also detected, including two T1PKS-NRPS hybrids, one T1PKS-indole, and one T1PKS-terpene.

The bio-active compounds produced by strain Aa22 (stemphol and stempholones A and B [19] are alkyl resorcinols. Type III polyketide synthases (T3PKSs) have been implicated in the biosynthesis of alkyl-resorcinols [46–48]. These enzymes use coenzyme A-bound substrates to form polyketide chains of varying length. They catalyze, within the same active site, the chain initiation, chain elongation, and cyclization reactions. Three different cyclization mechanisms have been described, giving rise to pyrone- quinolone- or resorcinol-polyketides [49,50]. The sole T3PKS identified by antiSMASH Fungi in the Aa22 genome (Fig 2) was further analyzed. A T3PKS from an endophytic *Fusarium incarnatum* isolate (GenBank accession KY780629.1), involved in the biosynthesis of resorcinol-like compounds [51], was used to BLAST search for similar regions in the Aa22 genome. A single significant match was identified within contig 16, where antiSMASH Fungi had predicted a T3PKS gene (Fig S1 in S1 File).

Pairwise sequence alignment showed 31.1% identity and 59.0% similarity between the *Fusarium* and *Stemphylium* proteins (File S1 in S1 File). In addition, functional annotations using InterProScan confirmed both *Fusarium* and *Stemphylium* proteins within the T3PKS family, identifying N-terminal (IPR001099) and C-terminal (IPR012328) chalcone/stilbene synthase domains (Table S3 in S1 File).

### Production of bio-active compounds

**Fig 3:**
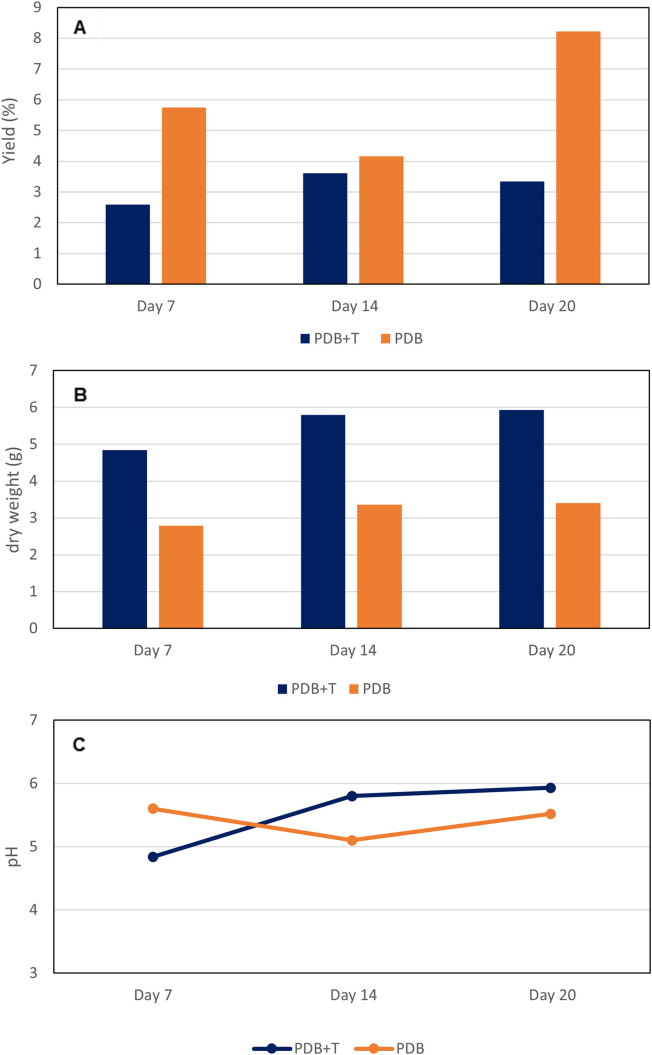
Submerged fermentation of strain Aa22 in PDB medium, with or without the addition of talcum powder. A: Solid extract yield; B: mycelium dry weight per fermentation; C: pH of the medium.

Fermentation yields in PDB are shown in Fig 3A. In liquid fermentation, the maximum yields were obtained in the absence of talcum powder. The solid fermentation showed a yield increase with time (2.85, 180.0 and 252.4 mg/100g after 7, 14 and 20 days, respectively).

Higher mycelium growth was observed in fermentations with PDB (Fig 3B). The pH (Fig 3C) remained slightly acidic with minimal variation from the initial pH of 5.1, averaging *ca.* 6. In the case of fermentations with talcum powder, the pH was slightly higher (initial 5.77) probably due to the magnesium ions alkalizing the medium.

**Fig 4:**
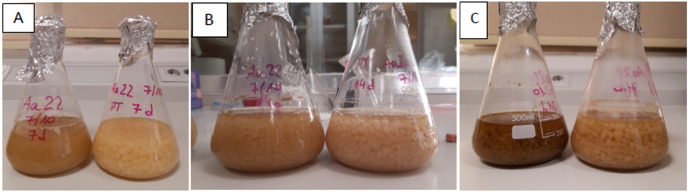
*Stemphylium* sp. Aa22 growth in fermentation flasks*.* PDB (left) and PDB plus talcum (right) after 7 (A), 14 (B), and 20 (c) days.

PDB cultures showed a characteristic, progressive darkening over time in PDB without talcum (Fig 4).

GC-MS analyses of extracts are presented in Table 1. Rice medium extracts were rich in fatty acids and derivatives, and their relative abundances changed with fermentation time. Major peaks were oleamide (10−18% with a peak at 14d), methyllinoleate (5−12% with a peak at 7d) and 9,12-hexadecadienoic acid, methyl ester (12–0.7% with a peak at 7d). No alkyl-resorcinols were detected. A mock rice extract had palmitic acid (26%), methyllinoleate (30%) and 8-heptadecenoic acid (28%) as the major components (Table S4 in S1 File), suggesting that the lipid-rich composition of the solid-state medium extracts derives from rice lipids and their metabolism.

**Table 1:** GC-MS analysis of the Aa22 extracts fermented in Rice, PDB or PDB plus talcum media (7, 14 and 20 days). Data are shown as percent abundance.

Retention time (min)	Compound	Rice			PDB			PDB + T	
		7 d	14 d	20 d	7 d	14 d	20 d	7 d	14 d
3.51	Phenylethyl alcohol				1.35	3.92		11.02	
4.22	4-(1-hydroxy-ethyl) -butanolactone				4.69			3.69	
7.34	1.2-Ethanediol				3.68	4.57		5.02	
7.66	N-Butylacetamide				5.13				
11.39	Acrylic acid tetradecanyl ester	1.10	2.79	1.33	1.35	1.12	0.41	5.06	6.73
14.95	Methyl palmitate	4.56	2.84	0.80	1.79				
17.41	9,12-Hexadecadienoic acid, methyl ester	12.09	2.24	0.76					
17.50	Methyloleate	9.10	1.97	0.97					
17.94	Methyllinoleate	12.37	7.23	5.20					
18.58	Palmitamide	1.22	3.26	1.65					
20.88	Oleamide	9.87	18.82	12.69	1.73	1.85		13.91	17.94
20.96	43/179/139/109/95/135/137/55/123/205/243				13.95	14.65	28.07		
21.04	Stempholone A				9.56	11.30	22.52		
21.44	3α-Hydroxy-manool				2.54		4.25		
24.37	190/43/107/137/95/245/81/121/55/109/209/227				8.16	20.88	16.76		
24.7/29.6	n-Hexatriacontane				0.97			8.15	3.27
26.80	Viridiflorol				3.61		6.57		
29.79	1-Eicosanol								5.07

PDB extracts contained, especially at early times, low molecular weight volatiles (<200 g.mol^-1^) such as phenylethyl alcohol (0–4%, peak at 14d), 4-(1-hydroxy-ethyl)-butanolactone (0–4.7%, at 7d), 1.2-ethanediol (0–4.6%, at 7d) and N-butylacetamide (0–5.1%, at 7d) (Table 1). Two unknown compounds were present at retention times of 20.96 min (28–14% with a peak at 21d) and 24.37 min (8–21% with a peak at 14d), together with stempholone A (9.5–22.5% with a peak at 20d). Comparison of the MS spectra of these two unknown compounds (RT 20.96, 24.37 min) with the targeted alkyl-resorcinols (stempholone A, stemphol and stempholone B) showed that one (RT 20.96 min) could be another hydroxylated derivative of stempholone A, and therefore a positional isomer of stempholone B, based on the common mass fragments (139, 43, 55, 69, 107, 109, 169) with an additional ion peak at *m/z* 179 that could be formed by the loss of [M^+^-C_4_H_9_O + H_2_O]. The second compound (RT 24.37 min) could be related to stemphol, based on the presence of a similar fragmentation pattern (fragments 190, 164, 137, 107, 121) (File S2 in S1 File). However, an unequivocal elucidation of their structures would require purification of these compounds, followed by a structural determination based on NMR spectroscopic techniques.

The presence of talcum powder (PDB + T) altered the extract composition, with higher levels of phenylethyl alcohol at 7 days (11%) and accumulation of oleamide (14–18% with a peak at 7d) and other long-chain lipids. Probably as a result, the PDB + T extract at 21 days could not be analyzed due to its low solubility in dichloromethane (Table 1). No detectable levels of stempholone A, or the related unknown metabolites (RT 20.96, 24.37 min), were found in PDB + T extracts.

**Fig 5:**
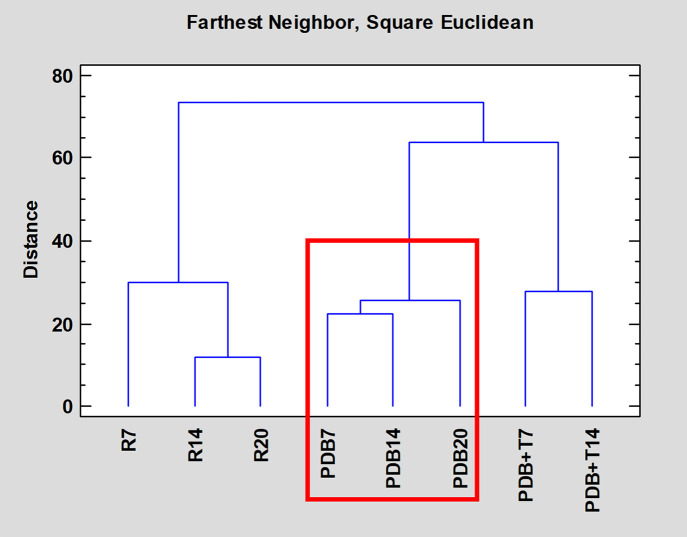
Dendrogram grouping of Aa22 extracts based on their chemical composition. Extracts containing Stempholone A are framed in red.

Fig 5 shows the composition-based dendrogram grouping of extracts. Two main groups were observed, matching the fermentation methods (solid and liquid). Additionally, two subgroups are observed for the liquid media (PDB and PDB + T). The PDB extracts were the only ones producing previously described bioactive compounds (stempholone A).

## Discussion

Whole genome sequencing and taxonomic profiling confirmed that isolate Aa22 represents a strain of the species *Stemphylium*, very close to *S. lycopersici*. However, given that some *S. solani* and *S. lycopersici* strains exhibit 100% identity in the ITS sequences originally used to taxonomically place strain Aa22 [19] and that no *S. solani* genome sequences are available, adscription of Aa22 to either species must remain uncertain. Both species are similar, and misclassification of strains has been reported as common [52]. *S. lycopersici* and *S. solani* are common plant pathogens usually isolated from diseased plants. Strain Aa22 was isolated as an endophyte from a healthy wormwood plant [19]. It is therefore noteworthy that its genome sequence is highly similar to those of pathogenic *S. lycopersici* isolates (Fig 1), suggesting that minor differences exist at the genomic level between pathogenic and endophytic strains. In fact, besides strain Aa22, several endophytic isolates of both *Stemphylium* species have been isolated [53–56] suggesting that the endophytic lifestyle could be common among members of these species.

Aa22 has been previously reported as the producer of bioactive alkyl resorcinols (stempholones A and B and stemphol, antifeedants against the aphid *Myzus persicae*) [19], whose biosynthetic pathways must be related based on their similar carbon backbone. Fungal biosynthetic pathways are complex, both in structure and in regulation [57]. This complexity makes it difficult to associate specific genes or gene clusters with the production of certain metabolites during genome annotation [58]. Regarding alkyl resorcinols, few T3PKSs specifically involved in their synthesis have been characterized [51]. The identified Aa22 T3PKS shared the conserved domains identified in the *F. incarnatum* enzyme that was experimentally shown to synthesize alkyl resorcinols [51], suggesting a related function in polyketide biosynthesis. However, with only 31.1% sequence identity and 46.0% similarity, structural and functional differences are expected between these two enzymes, which could result in the production of different related metabolites [59]. Given that only one T3PKS coding region was identified in the Aa22 genome, it is likely that its enzyme product plays a key role in the biosynthesis of stemphol, and by extension, their derivatives stempholones A and B. Heterologous expression and/or future transcriptomic and mutagenesis studies will help clarify this role. Additionally, elucidating its regulatory mechanisms will provide insights into pathway activation, potentially enabling targeted induction strategies to enhance metabolite production as has been done in other studies [60].

The production of bio-active compounds by Aa22 cultures was linked to culture conditions. In this study, solid-state fermentation on rice produced extracts that contained none of the previously described bioactive compounds [18] and were instead rich in lipids derived from the rice substrate. This may be due to the instability associated with using rice as a culture medium. In general, solid media are chemically complex and more difficult to control [23,61]. Factors such as particle size can affect productivity [61], and even the rice variety can significantly alter production patterns, influencing both yield and gene expression [62]. In turn, liquid fermentation in PDB medium resulted in higher extract yields with production of stempholone A along with two unidentified metabolites that increased with time. Addition of talcum powder (particle enhanced fermentation) decreased mycelium weight yield, an effect that had been observed in other filamentous fungi [63]. For Aa22, fermentation yields also decreased with the addition of talcum powder. Talcum is a good adsorbent of molecules, with applications as a clarifier [64]. This property may explain reduced extract yields. A different effect has been reported for an endophytic strain of *Phyllosticta capitalensis* [65] where talcum powder enhanced the production of meroterpenes, such as guignardone A and guignarenone B, at specific time points during fermentation, while the overall production of bioactive dioxolanones did not show significant improvements compared to the control. The effects of talcum powder on fermentation have also been linked to particle size. For instance, microparticles with a diameter of 40 μm improved the production of 2-phenyletanol by a strain of *Aspergillus terreus*, whereas smaller particles (10 μm) and iron oxide improved production of 2-phenyletanol and 6-pentyl-α-pyrone [63]. Conversely, small talcum powder diameter (5 μm) resulted in the lowest polysaccharide yields among tested materials [66]. These findings indicate that MPEC can significantly affect fermentations, either positively or negatively, across different organisms and metabolites. For Aa22, further research on MPEC is needed, either varying talcum powder diameter or using additional fermentation enhancers, such as bentonite or glass wool, as tested in other studies [65].

## Conclusions

The genome of *Stemphylium* sp. Aa22, an *Artemisia absinthium* endophytic strain previously described as a producer of the insect anti-feedant, alkyl-resorcinols stemphol and stempholones A and B, has been sequenced and analyzed. Genome-based taxonomic profiling placed strain Aa22 close to *S. lycopersici*. Thirty-eight BGCs, potentially responsible for biosynthesis of secondary metabolites, were detected. Among them, a sole type III polyketide synthase, potentially responsible for the biosynthesis of alkyl-resorcinols was identified.

Production of alkyl-resorcinols was strongly dependent on culture conditions. Solid fermentation on rice mainly produced lipid-based compounds, while fermentation in liquid PDB medium yielded the target compound stempholone A and two unknown, possibly related compounds. In contrast, Micro-Particle Enhanced Cultivation (MPEC) adding talcum powder to PDB (PDB + T) resulted in a lower diversity of metabolites compared to PDB, with a higher proportion of lipid-derived compounds.

Further optimization of culture medium composition and fermentation conditions is required to maximize the biosynthetic potential of strain Aa22 and facilitate its scale-up, thereby enabling the identification of the two unknown compounds observed. Additionally, a transcriptomic and mutant validation of the putative T3PKS as the cluster involved in the production of *Stemphylium* sp. Aa22 bioactive compounds will facilitate its biotechnological application.

## Supporting information

S1 File Zip file containing Tables S1-S4, Figure S1, and Files S1-S2. (RAR)
